# Population Pharmacokinetics of Pyronaridine in Pediatric Malaria Patients

**DOI:** 10.1128/AAC.02004-15

**Published:** 2016-02-26

**Authors:** Amal Ayyoub, Janthima Methaneethorn, Michael Ramharter, Abdoulaye A. Djimde, Mamadou Tekete, Stephan Duparc, Isabelle Borghini-Fuhrer, Jang-Sik Shin, Lawrence Fleckenstein

**Affiliations:** a College of Pharmacy, University of Iowa, Iowa City, Iowa, USA; b Centre de Recherches Médicales de Lambaréné, Hôpital Albert Schweitzer, Lambaréné, Gabon; c Institut für Tropenmedizin, Universität Tübingen, Tübingen, Germany; d Department of Medicine I, Division of Infectious Diseases and Tropical Medicine, Medical University of Vienna, Vienna, Austria; e Malaria Research and Training Center, Department of Epidemiology of Parasitic Diseases, Faculty of Pharmacy, University of Science, Techniques and Technologies of Bamako, Bamako, Mali; f Medicines for Malaria Venture, Geneva, Switzerland; g Shin Poong Pharmaceuticals Co., Ltd., Seoul, Republic of Korea

## Abstract

Pyramax is a pyronaridine (PYR)-artesunate (PA) combination for the treatment of uncomplicated malaria in adult and pediatric patients. A granule formulation of this combination is being developed for treatment of uncomplicated P. falciparum and P. vivax malaria in pediatric patients. The aims of this study were to describe the pharmacokinetics of PYR using a total of 1,085 blood PYR concentrations available from 349 malaria patients younger than 16 years of age with mild to moderate uncomplicated malaria and to confirm the dosing regimen for the pediatric granule formulation. Nonlinear mixed-effects modeling using NONMEM software was used to obtain the pharmacokinetic and inter- and intraindividual variability parameter estimates. The population pharmacokinetics of PYR were described by a two-compartment model with first-order absorption and elimination. Allometric scaling was implemented to address the effect of body weight on clearance and volume parameters. The final parameter estimates of PYR apparent clearance (CL/*F*), central volume of distribution (*V*_2_/*F*), peripheral volume of distribution (*V*_3_/*F*), intercompartmental clearance (*Q*/*F*), and absorption rate constant (*K_a_*) were 377 liters/day, 2,230 liters, 3,230 liters, 804 liters/day and 17.9 day^−1^, respectively. Covariate model building conducted using forward addition (*P* < 0.05) followed by backward elimination (*P* < 0.001) yielded two significant covariate-parameter relationships, i.e., age on *V*_2_/*F* and formulation on *K_a_*. Evaluation of bootstrapping, visual predictive check, and condition number indicated that the final model displayed satisfactory robustness, predictive power, and stability. Simulations of PYR concentration-time profiles generated from the final model show similar exposures across pediatric weight ranges, supporting the proposed labeling for weight-based dosing of Pyramax granules. (These studies have been registered at ClinicalTrials.gov under registration no. NCT00331136 [phase II study] and NCT00541385, NCT00403260, NCT00422084, and NCT00440999 [phase III studies]. The most recent phase III study was registered at pactr.org under registration no. PACTR201105000286876.)

## INTRODUCTION

Globally, an estimated 3.2 billion people are at risk of malaria. WHO estimates that 198 million cases of malaria and 584,000 malaria deaths occurred in 2013. Most cases (80%) and deaths (90%) occurred in Africa, and most deaths (78%) were in children under 5 years of age (1). Young children in Africa are disproportionately affected by malaria, and specific child-friendly antimalarial formulations are needed to meet their special needs, as it has been shown that pediatric drug formulations improve the tolerability of antimalarial drugs in young children (2).

Pyronaridine (PYR) is a benzonaphthyridine-derived antimalarial agent that has demonstrated efficacy against parasite strains resistant to other antimalarials, including amodiaquine and chloroquine (3, 4). Pyronaridine tetraphosphate has been coformulated in a 3:1 ratio with the artemisinin derivative artesunate to yield an oral artemisinin-based combination therapy for the treatment of acute uncomplicated Plasmodium falciparum and Plasmodium vivax malaria in children and adults. The tablet formulation of the oral fixed-dose combination product containing pyronaridine and artesunate (PA [Pyramax]) in the ratio of 3:1 was the first antimalarial to be granted a positive scientific opinion from the European Medicines Agency (EMA) under Article 58, in February 2012. This once-daily, 3-day treatment is indicated for acute, uncomplicated P. falciparum and blood-stage P. vivax malaria in adults and children over 20 kg. A granule formulation of this combination is being developed for treatment of uncomplicated P. falciparum and P. vivax malaria in pediatric patients <20 kg. To date, this combination has shown a favorable efficacy and adverse effect profile (5–9). The fixed-dose pyronaridine-artesunate combination showed high levels of efficacy of 99.2% in 749 Asian and African patients (95% confidence interval [CI], 98.3 to 99.7) (8) and 99.2% (95% CI, 98.7 to 99.9) in children and adults with uncomplicated falciparum malaria (9). The efficacy of Pyramax was high in many randomized clinical trials (6–10) and met the requirements for use as first-line therapy in an integrated analysis of individual patient data from six randomized clinical trials (6).

Pyronaridine is a lipophilic compound which displays extensive distribution and slow *in vivo* elimination, with a typical half-life of 13.2 days in adults with malaria and 11.3 days in healthy adults (3). Pyronaridine is effective against the erythrocytic stage of several species of malaria parasites (3). The mechanism of action is similar to that of chloroquine with regard to inhibition of β-hematin formation, which leads to disruption of hematin detoxification in malaria parasites. Pyronaridine is known to concentrate in red blood cells, and thus, blood is the preferred biological matrix for pharmacokinetics studies. A mass balance and metabolite characterization study was conducted in six healthy male adults administered a single oral dose of 720-mg pyronaridine tetraphosphate with 800 nCi of radiolabeled [^14^C]pyronaridine (11). Nine primary and four secondary metabolites of pyronaridine were identified. This study revealed that pyronaridine and its metabolites are eliminated by both the urinary and fecal routes over an extended period of time and that multiple, varied pathways characterize pyronaridine metabolism (11).

In the current analysis, the population pharmacokinetics of pyronaridine was evaluated in pediatric patients with uncomplicated malaria participating in one phase II and five phase III clinical studies with PA dosed in the range of 6:2 mg/kg of body weight to 12:4 mg/kg for pyronaridine and artesunate, respectively. We also investigated the influence of several potential covariates on the pharmacokinetics of pyronaridine. An extensive blood sampling scheme was utilized in the phase II study and a sparse sampling scheme in the phase III studies.

## MATERIALS AND METHODS

### Study designs and blood sampling.

The blood concentrations of pyronaridine from one phase II study (SP-C-003-05) and five phase III studies (SP-C-004-06, SP-C-005-06, SP-C-006-06, SP-C-007-07, and SP-C-013-11) with PA were pooled for purposes of population pharmacokinetics analysis. All subjects in these trials provided written informed consent for their participation. All studies were approved by the local ethics committee. The phase II and four of the phase III studies were registered in clinicaltrials.gov under the following registration numbers: NCT00331136 for the phase II study and NCT00541385, NCT00403260, NCT00422084, and NCT00440999 for the phase III studies. The most recent phase III study was registered in pactr.org under the registration number PACTR201105000286876. An overview of the study design of each clinical study is summarized as follows.

### Phase II trial.

The phase II study SP-C-003-05 was aimed to assess the safety, tolerability, and pharmacokinetics of the 3-day regimen of PA in tablet and granule formulations for the treatment of uncomplicated P. falciparum malaria in pediatric patients in Gabon. For PA tablets, patients were sequentially assigned to one of 3 treatment groups. For granule formulations, the PA dose of 9:3 mg/kg was used. Blood samples were collected immediately before each dose and at 0.5, 1, 1.5, 2.5, 4, 8, and 12 h and 3, 7, 14, and 21 days after the first dose.

### Phase III trials.

All studies in phase III were comparative, randomized studies to assess the efficacy and safety of PA with those of other antimalarial drugs. SP-C-004-06 and SP-C-005-06 were comparative studies to assess the efficacy and safety of PA (180:60 mg) with those of mefloquine (250 mg) plus artesunate (100 mg) and with those of artemether-lumefantrine (20:120 mg) (Coartem), respectively, in children and adults with uncomplicated P. falciparum malaria. SP-C-006-06 was conducted to compare the safety and efficacy of PA (180:60 mg) with those of chloroquine (155 mg) in patients with acute P. vivax malaria. SP-C-007-07 was a comparative, randomized study to assess the safety and efficacy of a granule formulation (60:20 mg) (pediatric Pyramax) versus the safety and efficacy of crushed Coartem tablets in infants and children with acute uncomplicated P. falciparum malaria. SP-C-013-11 was a comparative, randomized clinical study to assess the safety and efficacy of repeated administration of pyronaridine-artesunate, dihydroartemisinin-piperaquine, artemether-lumefantrine, or artesunate-amodiaquine over a 2-year follow-up period in children and adults with acute uncomplicated Plasmodium sp. malaria. For SP-C-004-06, SP-C-005-06, and SP-C-006-06, PA was given according to body weight: 20- to ≤25-kg patients received one tablet, 26- to <45-kg patients received two tablets, ≥45- to <65-kg patients received three tablets, and ≥65- to 90-kg patients received four tablets. For SP-C-007-07, PA was given as follows: ≥5- to <9-kg patients received one sachet, 9- to <17-kg patients received two sachets, and 17- to <25-kg patients received three sachets. For SP-C-013-11, PA was given as follows: ≥5- to <8-kg patients received one sachet, 8- to <15-kg patients received two sachets, and 15- to <20-kg patients received three sachets. For all phase III studies, one or two blood samples were collected at two different time points (between day 0 and day 3 and between day 4 and day 42).

### Sample handling and analysis.

Venous blood samples (1 ml for small children) were drawn into heparinized tubes (Vacutainer tubes). Two approximately equal-volume aliquots of the blood were transferred to screw-cap cryovials (Nalgene 50000012) and frozen at or below −80°C in a laboratory freezer. They were later shipped separately, frozen on dry ice, via air express to the Clinical Pharmacokinetics Laboratory at the College of Pharmacy, University of Iowa. All samples were stored at −80°C until drug analysis was performed. For all studies, the blood concentrations of pyronaridine were quantified by a validated liquid chromatographic mass spectrometer (LC-MS) (12–14). Chromatographic analysis was carried out on a Shimadzu model 2010A liquid chromatography and mass spectrometer (Shimadzu, Columbia, MD, USA). The mobile phase was delivered using a Shimadzu LC-10AD VP solvent delivery system (solvent A was 2 mM perfluorooctanoic acid, and solvent B was acetonitrile). Sample injections were made using a Shimadzu SIL-10AD VP auto injector. The high-performance liquid chromatography (HPLC) column used for chromatographic analysis was a Phenomenex Gemini column (150 by 2.00 mm, 5-μm particle size, C_18_, 110-Å pore size). The column was housed in a Shimadzu CTO-10AS VP column oven. Chromatographic data were analyzed using Lab Solutions LCMS Solution software, version 3.30.268. The calibration curve had a lower limit of quantitation of 5.7 ng/ml and was linear over the range of 5.7 to 855 ng/ml. Samples with a concentration higher than 855 ng/ml were diluted so that the concentration fell within the range of the calibration curve. The intraday percent coefficients of variation for pyronaridine samples (11.4, 285, and 760 ng/ml) were 11.1%, 4.8%, and 2.2%, respectively. The percent coefficients of variation of interday analyses of pyronaridine samples (11.4, 285, and 760 ng/ml) were 15.9, 9.7, and 7.8%, respectively.

### Population pharmacokinetic and statistical analyses.

Nonlinear mixed-effect model building was conducted using NONMEM software version VII, level 2.0 (2011; ICON Development Solutions), as implemented on a Windows XP operating system (Microsoft Corporation, Redmond, WA) with a G95 Fortran compiler. NONMEM output was generated using PDx-Pop version 5.0 (2011; ICON Development Solutions) and Xpose version 4.3.0 (2010; Uppsala University, Uppsala, Sweden). Graphical plots were produced using TIBCO Spotfire S+ version 8.1 (2008; TIBCO Software, Inc.) and R 2.10.1 (2010; The R Foundation for Statistical Computing). Blood pyronaridine concentrations were natural log transformed before the analysis, and the dose of pyronaridine tetraphosphate was converted to pyronaridine base by multiplying by a factor of 0.57 prior to modeling. All models were fitted using the first-order conditional estimation (FOCE) method. Model selection was based on the plausibility and precision of the parameter estimates, a comparison of minimum objective function value (MOFV), which is equal to minus twice the log likelihood function, Akaike information criterion (AIC), which is equal to MOFV plus two times the number of parameters, and condition number, which is defined as the ratio of the largest Eigen value to the smallest Eigen value, as well as visual inspection of diagnostic plots.

### Base model development.

Based on the exploratory data analysis, a two-compartment pharmacokinetic model with first-order absorption and elimination from the central compartment was initially fitted to pyronaridine data. The model was parameterized in terms of *K_a_* (absorption rate constant), CL/*F* (apparent clearance), *V*_2_/*F* (central volume of distribution), *V*_3_/*F* (peripheral volume of distribution), and *Q*/*F* (intercompartmental clearance). The effect of body weight on the apparent clearance and volume of distribution terms was addressed using an allometric scaling approach with exponents of 1 and 0.75 on volume and clearance parameters, respectively. The interindividual variability (IIV) of the pharmacokinetic parameters was modeled as following a log-normal distribution, as follows: *P_i_* = *P*_pop_ · exp(η_*i*_), where *P_i_* is the estimated parameter value for individual *i*, *P*_pop_ represents the typical population estimate for the parameter, and η_*i*_ is the deviation of *P_i_* from *P*_pop_. The η random effects were assumed to be independent and normally distributed with zero mean and variance of ω^2^. The magnitude of IIV was expressed as the percent coefficient of variation. Residual variability (RV) was modeled using an additive model, as follows: ln *C_ij_* = ln *C*_pred,*ij*_ + ε_*ij*_, where *C_ij_* and *C*_pred,*ij*_ represent the *j*th observed and model-predicted pyronaridine concentrations, respectively, for individual *i* and ε_*ij*_ denotes the additive residual random error for individual *i* and observation *j*. The ε random effects were assumed to be independent and symmetrically distributed with zero mean and variance of σ^2^. Attempts were made to incorporate a full variance-covariance matrix; however, successful convergence with plausible parameter estimates could not be attained.

### Covariate model building.

The influence of subject-specific covariates on the estimated pharmacokinetic parameters was evaluated. Correlations between tested covariates were examined by graphical analysis. Plots of the individual empirical Bayes estimates of the parameters versus covariates were generated to visualize potential relationships. Covariate selection was based on the generalized additive model (GAM) as implemented in Xpose, the graphic explorations, and physiologic plausibility. The covariates that were used in population pharmacokinetic model building included the following: baseline creatinine clearance, baseline alanine aminotransferase (ALT), gender, age (as both a continuous and categorical covariate), formulation, and baseline hemoglobin (Hgb). Covariates identified as being important were included in the mixed-effects model by applying a procedure of forward addition (*P* < 0.05) followed by backward elimination (*P* < 0.001). Depending on the graphical exploration of the relationship between a covariate and a pharmacokinetic parameter, the effect of the covariate on the parameter was tested with a linear function, a power function, or an exponential function. All continuous covariates were scaled or centered on the median values so that the population estimates represent those of an average patient. The equations used were *P* = (θ_1_ + θ_2_) · (COV − COV_median_) for the linear function, *P* = θ_1_ · (COV/COV_median_)^θ2^ for the power function, and *P* = θ_1_ · exp[θ_2_ · (COV − COV_median_)] for the exponential function, where θ_1_ represents the parameter estimate (*P*) of an individual with the median value of the covariate (COV_median_) and θ_2_ is a factor describing the correlation between the covariate (COV) and the parameter. The influences of categorical covariates on the parameter were modeled using an additive relationship, as follows: *P* = (θ_3_ + θ_4_) · COV, where θ_3_ represents the parameter value in subjects with the categorical covariate coded as 0 and θ_4_ is the additional change in the parameter in subjects with the categorical covariate coded as a value other than 0.

Statistically significant improvement in the MOFV, improvement in the precision of the parameter estimate (relative standard error), and reductions in interindividual and residual variability were used to determine the importance of the covariates as predictors.

Discrimination between two nested models was based on the improvement of the MOFV, which is equal to twice the negative log likelihood of the data. A decrease in the MOFV (which approximately follows the χ^2^ distribution, where the degree of freedom is the difference in the number of estimated parameters) of 3.84 units was considered statistically significant (*P* < 0.05) for the addition of one parameter to a candidate model. The addition of covariates into the model was continued until a decrease of 3.84 could not be reached any longer. This was defined as the fully parameterized population pharmacokinetic model. To determine whether all the covariates included in the fully parameterized population pharmacokinetic model continued to provide significant influence on the population model, the covariates included in the full model were sequentially removed and the resulting reduced model evaluated to determine whether there was significant model degradation. The significance of the covariate was tested using the nested model criteria at a more stringent *P* value of 0.001, resulting in a change in OFV of 10.83, to avoid false positives. The elimination process was repeated until all remaining covariates in the model were significant.

### Model evaluation.

The goodness-of-fit of the models was assessed using diagnostic plots of population predicted versus observed dependent variable (PRED versus DV), individual predicted versus observed dependent variable (IPRED versus DV), conditional weighted residuals versus the predicted dependent variable (CWRES versus PRED), and conditional weighted residuals versus time (CWRES versus TIME). The standard errors and 95% confidence intervals for the parameter estimates in the final model were estimated using the nonparametric bootstrap approach. One thousand bootstrap data sets were generated by repeated random sampling with replacement from the NONMEM input data file, and the final NONMEM model was fitted to the bootstrap data sets. The mean and 95% confidence intervals for the population pharmacokinetic parameters were calculated and compared with the estimates from the original data set. The bootstrap 95% confidence intervals were calculated based on the percentile of the empirical distribution of the estimated parameters from the bootstrap runs. A visual predictive check was performed to evaluate the predictive ability of the final model. A thousand virtual observations at each sampling time point were simulated using the final model and its parameter estimates. The observed data were then plotted with the 5th, 50th, and 95th percentiles of the simulated data. The percentage of observations outside the 90% prediction interval was also calculated. Condition number was calculated as a measure of the stability of the model. The condition number is defined as the ratio of the largest Eigenvalue to the smallest Eigenvalue and was calculated by using the PRINT = E option on the $COV statement of the NONMEM control stream. The condition number should ideally be ≤1,000, as a condition number exceeding 1,000 is indicative of ill conditioning of the model.

### Simulations.

Monte Carlo simulations from the final pharmacokinetic value and interindividual variability estimates model were performed using NONMEM. Simulated pyronaridine concentration-time profiles were conducted for two weights in each of the three granule PA dosing groups; the two weights were selected to represent the lower and upper limit weights for each dosing group (i.e., 5, 7, 8, 14, 15, and 19 kg). Five hundred concentration-time profiles were simulated for each representative weight. The simulations were used to obtain the 5th, 50th, and 95th percentile concentrations corresponding to the administration of a regimen of three once-daily doses of the granule formulation. As age was included as a covariate on *V*_3_/*F* in the final model, the ages for which each weight represented the approximate median weight were obtained from the WHO growth standards (15). As the growth standards differ between males and females, the gender-specific ages were averaged. For the 5- and 7-kg simulated patients, the minimum proposed age for PA granule labeling (6 months) was utilized.

In addition, to support the dosing scheme in children weighing less than 20 kg and more than 8 to 10 kg and to show consistent systemic exposure of pyronaridine based on the pediatric dosing scheme compared to the systemic exposure in adult patients, simulations for pyronaridine exposure (area under the curve from zero to infinity [AUC_0–∞_]) were conducted in NONMEM for hypothetical patients with weights ranging from 5 kg to 19 kg for the pediatric population and from 20 kg to 60 kg for the adult population. The simulations were used to obtain the area under the curve (ln AUC_0–∞_) values corresponding to the administration of a pyronaridine-artesunate regimen of three once-daily doses of the tablet and granule formulations for the adult and pediatric patients, respectively.

## RESULTS

### Demographic data.

Data from one phase II and five phase III clinical studies were pooled. Among 1,257 blood pyronaridine levels from 351 pediatric malaria patients collected to determine pyronaridine concentrations, approximately, 13.5% (165 observations) of the samples were below the lower limit of quantitation and 0.24% (3 observations) were identified as outliers and were excluded from the analysis. A total of 1,091 blood pyronaridine concentration values collected from 347 subjects were available for the analysis. Table 1 summarizes the numbers of subjects and observations and demographic and clinical characteristics of the population in each clinical study. This pediatric population had a median age and median weight of 7 years and 20 kg, respectively. The numbers of males and females in this population were comparable (47.6% and 52.4% for females and males, respectively). Most of the patients received PA as a tablet formulation (67.0%).

**TABLE 1:** A summary of study data, patient demographics, and covariates included in the analysis

Characteristic^*a*^	Value for indicated trial(s)						
	Phase II, SP-C-003-05	Phase III					All studies combined
		SP-C-004-06	SP-C-005-06	SP-C-006-06	SP-C-007-07	SP-C-013-11	
No. of subjects	57	40	143	9	83	17^*b*^	349
Total no. of observations	702	79	272	17	150	32	1,252
No. (%) of observations excluded as <LOQ	14 (2)	21 (26.6)	75 (27.6)	6 (35.3)	49 (32.7)	0 (0)	165 (13.2)
No. (%) of observations excluded as outliers	1 (0.14)	0 (0)	1 (0.4)	0 (0)	1 (0.7)	0 (0)	3 (0.24)
No. (%) of observations included in the analysis	688 (98)	58 (73.4)	196 (72.1)	11 (64.7)	100 (66.6)	32	1,085 (86.7)
Median age (range) (yr)	5 (2–14)	11 (5–15)	9 (5–15)	14 (9–15)	5 (0.6–10)	2.08 (0.51–3.58)	7 (0.51–15)
Median wt (range) (kg)	16.2 (10–36.4)	20.9 (20–46.4)	26.6 (20–56.2)	31.5 (20–46.7)	17 (9–24.3)	11.3 (6.8–17.1)	20 (6.8–56.2)
Median BMI (range) (kg/m^2^)	15 (12.8–17.6)	15.1 (11–20)	16.6 (11.5–26.5)	15.8 (13.6–19.2)	15 (6–23)	14.6 (12.9–17.4)	15.4 (6–26.5)
Baseline parasite count (range) per μl	6,304 (1,072–174,241)	10,559 (1,201–92,500)	13,088 (1,000–93,923)	8,632 (1,193–51,947)	14,400 (153–188,488)	37,080 (104–157,860)	9,260 (104–188,488)
Median baseline hemoglobin (range) (g/dl)	102 (74–129)	110 (82–130)	115 (84–208)	113 (93–135)	100 (80–123)	99 (74–120)	105 (74–208)
No. (%) female	28 (49.1)	19 (47.5)	78 (54.5)	3 (33.3)	45 (54.2)	10 (58.8)	183 (52.4)
No. (%) male	29 (50.9)	21 (52.5)	65 (45.5)	6 (66.7)	38 (45.8)	7 (41.12)	166 (47.6)
No. of subjects with AST >1.5 ULN	36	5	6	0	3	5	55
No. of subjects with ALT >1.5 ULN	5	1	2	0	0	1	9

a LOQ, limit of quantitation; BMI, body mass index; AST, aspartate transaminase; ALT, alanine aminotransferase; ULN, upper limit of normal.

b Two individuals in SPC-013-11 vomited and were excluded from the analysis.

### Population pharmacokinetic model.

A two-compartment model with first-order absorption and elimination from the central compartment described the observed data satisfactorily and was parameterized in terms of absorption rate constant (*K_a_*), apparent central volume of distribution (*V*_2_/*F*), apparent peripheral volume of distribution (*V*_3_/*F*), oral clearance (CL/*F*), and intercompartmental clearance (*Q*/*F*), where *F* is oral bioavailability. Allometric scaling was applied to address the effect of weight on clearance and volume parameters; specifically, body weight was centered on the median value of 20 kg and treated as a fixed covariate with exponents of 1 and 0.75 on volume and clearance parameters, respectively. According to the GAM, graphical exploration, and physiologic plausibility, the potential covariate-parameter relationships to be tested using forward addition and backward elimination were as follows: formulation and age on *K_a_*; age, creatinine clearance, gender, ALT value, and formulation on CL/*F*; age, gender, and Hgb on *V*_2_/*F*, and age and gender on *V*_3_/*F*. The full model from forward addition contained age on *V*_3_/*F* and formulation on *K_a_*. During stepwise backward elimination, both age on *V*_3_/*F* and formulation on *K_a_* resulted in a significant increase in the MOFV upon the removal of each covariate from the full model at the 0.001 significance level and in an improvement in the interindividual variability, and consequently, they were retained in the final model. After the inclusion of statistically significant covariates, the final parameter values for *K_a_*, CL/*F*, *V*_2_/*F*, *V*_3_/*F*, and *Q*/*F* were 17.9 day^−1^, 377 liters/day, 2,230 liters, 3,230 liters, and 804 liters/day. All parameters were estimated with acceptable precision. The IIV on *Q*/*F* was fixed to zero, as the estimates could not be obtained with good precision. The percent coefficients of variation of IIV on CL/*F*, *V*_2_/*F*, *V*_3_/*F*, and *K_a_* were 40.7%, 99.6%, 50.6%, and 65.8%, respectively. The mean elimination half-life of pyronaridine in pediatric malaria subjects was estimated to be 12.3 days. The final parameter estimates given by this model are summarized in Table 2.The relationships between significant covariates and pharmacokinetic parameters were described as follows: *V*_3_/*F* (liters) = [3,230 · (weight/20) · (age/7)0.624] · exp(η) and *K_a_* (hours) = [17.9 · (1 + formulation · 1.63)] · exp(η).

**TABLE 2:** Summary of the results obtained from the final model and the bootstrap analysis

Parameter^*a*^	Median value for^*b*^:				
	Estimate	%RSE	%CV	Bootstrap estimate (95% CI)	
CL/*F* (liters/day)	377	6.58		371.7	(302–435)
*V*_2_/*F* (liters)	2,230	6.59		2,212	(1,930–2,580)
*V*_3_/*F* (liters)	3,230	15.0		3,298	(2,360–4,710)
*Q*/*F* (liters/day)	804	11.2		831	(651–1,130)
*K_a_* (day^−1^)	17.9	11.7		17.7	(14.1–22.5)
θ_6_ age on *V*_3_/*F*	0.624	38.6		0.629	(0.224–1.05)
θ_7_ formulation on *K_a_*	1.63	37.8		1.86	(0.517–4.11)
IIV					
CL/*F*	0.166	26.7	40.7	0.160	(0.055–0.318)
*V*_2_/*F*	0.993	8.76	99.6	0.994	(0.805–1.170)
*V*_3_/*F*	0.256	48.4	50.6	0.265	(0.033–0.593)
*Q*/*F*					
*K_a_*	0.433	26.6	65.8	0.413	(0.194–0.669)
RV (additive error)	0.195	12.1		0.195	(0.15–0.244)

a For both of the derived parameters distribution half-life (*T*_1/2α_) and elimination half-life (*T*_1/2β_) for pediatric malaria patients, the mean value ± standard deviation was 0.9 ± 0.6. *F*, oral bioavailability; CL, clearance; *V*_2_, central volume of distribution; *V*_3_, peripheral volume of distribution; *Q*, intercompartmental clearance; *K_a_*, first-order absorption rate constant; IIV, interindividual variability; RV, residual variability.

b %RSE, relative standard error [(SE/mean) × 100%]; %CV, percent coefficient of variation [(SD/estimate) × 100%].

### Model evaluation.

The final population pharmacokinetic model was fitted repeatedly to 1,000 bootstrap samples. The percent convergence rate obtained from the bootstrap results was 85.9%. The mean parameter estimates obtained from the bootstrap replicates are summarized in Table 3. The final model provided estimates within the 95% confidence intervals obtained by 1,000 bootstrap runs. The bootstrap estimates are reasonably close to those obtained from the final model.

**TABLE 3:** Weights and ages used in pyronaridine simulations

Wt group	Wt (kg)	Age (yr)	No. of sachets	PP^*a*^ dose (mg)	Pyronaridine base dose (mg)	PP dose (mg/kg)
1	5	0.5	1	60	34.2	12
2	7	0.5	1	60	34.2	8.57
3	8	0.583	2	120	68.4	15
4	14	3	2	120	68.4	8.57
5	15	3	3	180	102.6	12
6	19	5	3	180	102.6	9.47

a PP, pyronaridine tetraphosphate.

**FIG 1:**
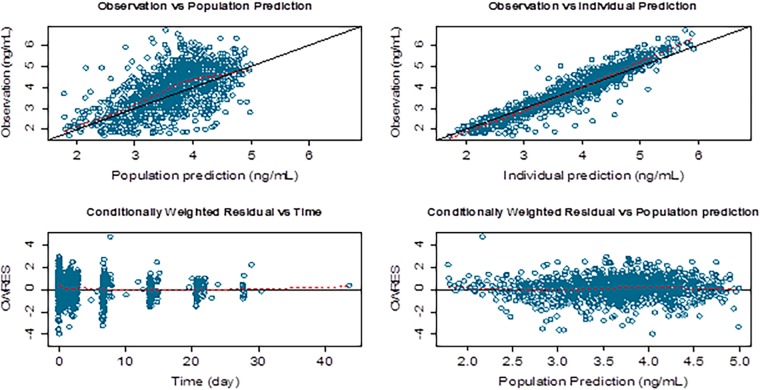
Goodness of fit plots of pyronaridine for the final model. The solid lines in the top panels are lines of identity. The broken lines are smoothing lines.

**FIG 2:**
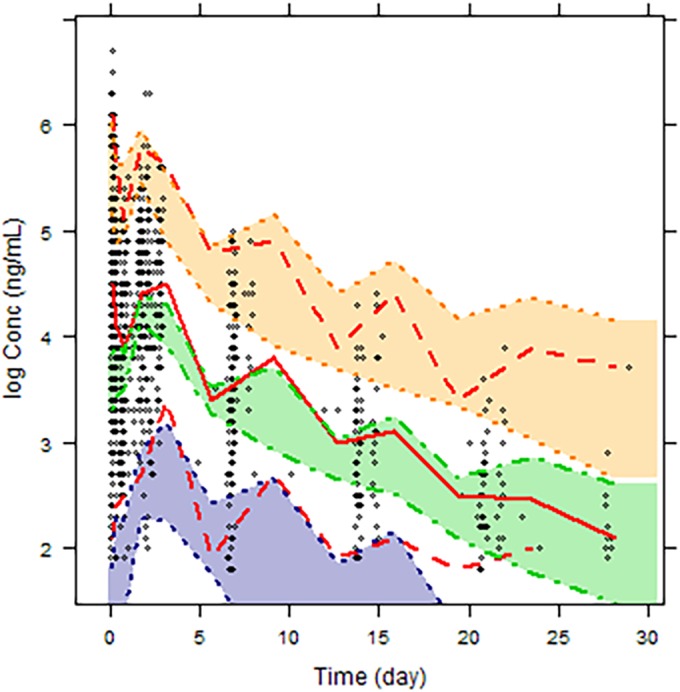
Visual predictive check of the final model. The diamonds represent the observed concentrations, the dashed red lines represent the 5th and 95th percentiles, and the solid red line represents the 50th percentile obtained from the simulations. The shaded areas and corresponding dashed lines represent the simulated data prediction intervals about the observed data percentiles.

**FIG 3:**
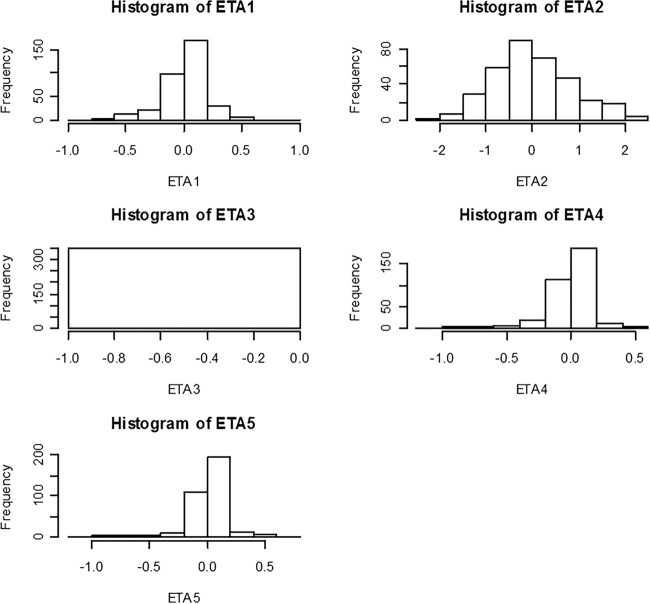
Distribution of interindividual variability for pyronaridine clearance from the central compartment (CL/*F*) (ETA1), volume of the central compartment (*V*_2_/*F*) (ETA2), intercompartmental clearance (*Q*/*F*) (ETA3), volume of the peripheral compartment (*V*_3_/*F*) (ETA4), and absorption rate constant (*K_a_*) (ETA5) for the final model.

Figure 1 shows goodness-of-fit plots of pyronaridine. Figure 2 shows the results of the visual predictive check for pyronaridine. Overall, the final model adequately described the observed concentrations. About 4.25% of the pyronaridine observations were not contained within the 95% prediction interval of the visual predictive check for pyronaridine using time; specifically, 2.03% and 2.22% were above and below the 95% prediction interval, respectively. The condition number of the final model was 36.7, indicating that the model was stable. The ETA (interindividual variability parameter) values had normal distribution and shrinkage values were 48.82%, 15.19%, 63.80%, and 71.60% for the CL/*F*, *V*_2_/*F*, *V*_3_/*F*, and *K_a_* parameters, respectively (Fig. 3).

### Simulations.

**FIG 4:**
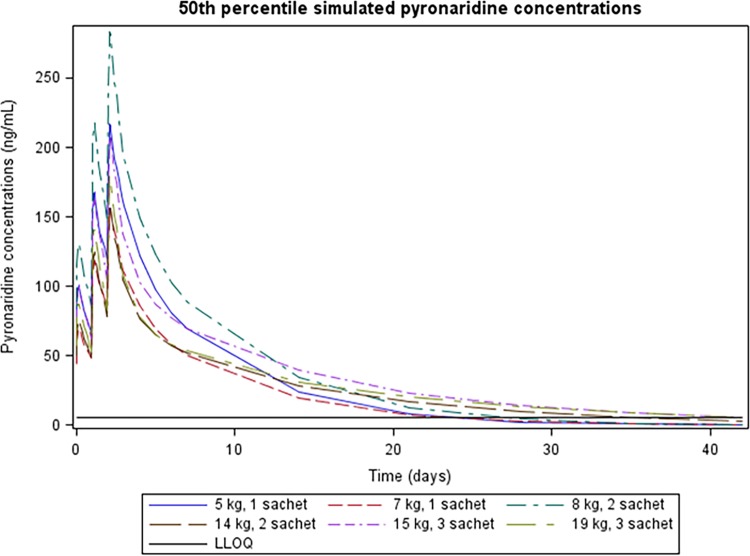
Fiftieth percentile simulated pyronaridine concentrations for a 5-kg, 6-month-old patient and a 7-kg, 6-month-old patient administered 1 sachet, an 8-kg, 7-month-old patient and a 14-kg, 3-year-old patient administered 2 sachets, and a 15-kg, 3-year-old patient and a 19-kg, 5-year-old patient administered 3 sachets.

Simulations were performed at the extremes of the weight range for each dosing group. The 50th percentile of simulated pyronaridine concentration-time profiles in the central compartment for each of the two representative weights in a given label dosing group for pediatric malaria-infected subjects are presented in Fig. 4.

**FIG 5:**
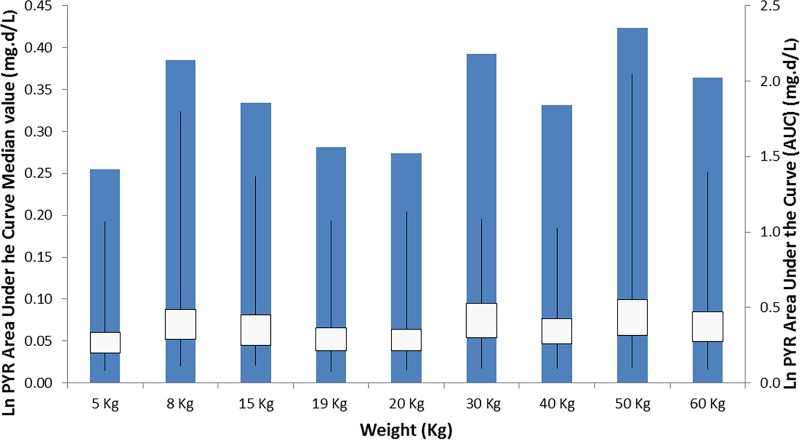
Simulated pyronaridine AUC_0–∞_ (mg · day/liter) versus weight (kg) for malaria-infected subjects after the administration of the tablet dosage form for those weighing ≥20 kg and the granule dosage form for those weighing <20 kg.

Simulated ln AUC_0–∞_ values for the granule dosage administration for subjects weighing <20 kg and the tablet dosage administration for subjects weighing ≥20 kg are presented in Fig. 5 to illustrate uniform exposure across the weight ranges for the proposed labeled dosing of granules and tablets.

## DISCUSSION

Pyronaridine has proven to be an effective partner drug in artemisinin-based combination treatment for malaria. A total of 1,085 blood pyronaridine concentration values collected from 347 subjects participating in one phase II and five phase III clinical studies with PA (either tablets or granule formulation) were included in the present analysis. The objective of the analysis was to describe the population pharmacokinetics of pyronaridine in pediatric malaria patients, with a particular focus on describing any formulation-specific effects associated with administration of the granule formulation.

The results from the population pharmacokinetic analysis showed that pyronaridine is well described by a two-compartment model with first-order absorption and elimination. A fixed-exponent allometric-scaling approach was used to incorporate the effect of body weight on volume and clearance parameters (16–20). The goodness-of-fit plots for the three age groups of <5 years, 6 to <12, and >12 years of age from the final model suggested a reasonable fit of the model to the data. All parameters were estimated with acceptable precision with relative standard error values of 15% or less on all fixed-effect parameters and less than 30% on variability parameters, with the exception of variability on intercompartmental clearance (*Q*/*F*) (relative standard error, 48.4%). The variability was most pronounced on central volume of distribution (99.6%). The parameter estimates obtained from the final model were close to those generated from 1,000 bootstrap runs, suggesting robustness of the model. The visual predictive check showed that the final population model adequately captured the majority of the data. Additionally, the model is stable as evidenced by the condition number. In this analysis, body size was considered a primary factor to describe variability in pharmacokinetics among children. The effect of body size was incorporated as a fixed covariate by using an allometric exponent that has a strong theoretical and empirical basis (20). The main physiological variables that showed significant influence on pyronaridine disposition were age on the peripheral volume of distribution (*V*_3_/*F*) and formulation on the absorption rate constant (*K_a_*). Pyronaridine is a highly basic compound that is sparingly soluble in water and distributes extensively into tissues represented by the peripheral compartment. The effect of age on *V*_3_/*F* with an exponent of 0.624 indicates an increase in the peripheral volume of distribution with age, a matter that could be attributed to potential age-related developmental changes of various processes, as well as changes in body composition involved in the disposition of pyronaridine. Developmental changes in transporter expression and tissue binding capacity could quite plausibly affect pyronaridine disposition, thereby contributing to the modeled age effect on the peripheral volume of distribution. It should be noted that this effect of age on peripheral volume of distribution does not influence overall pyronaridine exposure, which is dependent solely on CL/*F*.

The other significant covariate was formulation on *K_a_*, with an estimated proportional increase of 1.63 in the value of *K_a_* for the granule dosage form compared to the tablet dosage form. This effect could be confounded by the age of the pediatric population, since most of the subjects who were administered the granule dosage form were quite young.

Based on the simulations performed in this analysis, it appears that the proposed dosing strategy for the granule formulation is expected to result in similar exposure to pyronaridine for patients across the relevant weight range and in comparison to the adult population.

Due to the difficult nature of frequent blood sampling in malaria-infected and often anemic pediatric populations, with one observation during the period of day 0 to day 3, a small, clinically nonrelevant difference in the effect of formulation on the absorption rate constant might exist, caused by the relatively small number of data points available to characterize the absorption phase differences between the two formulations.

In conclusion, a two-compartment model with first-order absorption and elimination best described the pharmacokinetics of pyronaridine. Allometric scaling was used to incorporate the effects of body size into the pharmacokinetic estimation of the clearance and volume parameters. Age was identified as a significant predictor of pyronaridine peripheral volume of distribution, and formulation was a significant covariate on pyronaridine absorption rate constant. The final model is stable, predictive, and robust, with acceptable precision of parameter estimates. Furthermore, simulations generated from the final model support the proposed dosage regimen in PA granule labeling for pediatric malaria patients.
